# Impact of vaccine type on HIV-1 vaccine elicited antibody durability and B cell gene signature

**DOI:** 10.1038/s41598-020-69007-w

**Published:** 2020-08-03

**Authors:** Rohith Palli, Kelly E. Seaton, Michael S. Piepenbrink, John Hural, Paul A. Goepfert, Fatima Laher, Susan P. Buchbinder, Gavin Churchyard, Glenda E. Gray, Harriet L. Robinson, Yunda Huang, Holly Janes, James J. Kobie, Michael C. Keefer, Georgia D. Tomaras, Juilee Thakar

**Affiliations:** 1 Medical Scientist Training Program, University of Rochester School of Medicine and Dentistry, Rochester, NY USA; 2 Biophysics, Structural, and Computational Biology Program, University of Rochester School of Medicine and Dentistry, Rochester, NY USA; 3 Duke Human Vaccine Institute and Departments of Surgery, Immunology, and Molecular Genetics and Microbiology, Duke University Medical Center, Durham, NC USA; 4 Infectious Diseases Division, Department of Medicine, University of Alabama at Birmingham, Birmingham, AL USA; 5 Vaccine and Infectious Disease Division, Fred Hutchinson Cancer Research Center, Seattle, WA USA; 6 Perinatal HIV Research Unit, Faculty of Health Sciences, University of the Witwatersrand, Johannesburg, South Africa; 7 Bridge HIV, San Francisco Department of Public Health and Departments of Medicine, Epidemiology and Biostatistics, University of California, San Francisco, CA USA; 8 Aurum Institute, Johannesburg, South Africa; 9 South African Medical Research Council, Cape Town, South Africa; 10 GeoVax Labs, Inc., Smyrna, GA USA; 11 Public Health Sciences Division, Fred Hutchinson Cancer Research Center, Seattle, USA; 12 Department of Medicine, Infectious Diseases Division, University of Rochester School of Medicine and Dentistry, Rochester, NY USA; 13 Department of Microbiology and Immunology, University of Rochester, Rochester, NY 14620 USA; 14 Department of Biostatistics and Computational Biology, University of Rochester, Rochester, NY 14620 USA

**Keywords:** Adjuvants, DNA vaccines, Protein vaccines, Antibodies, Regulatory networks, HIV infections

## Abstract

Efficacious HIV-1 vaccination requires elicitation of long-lived antibody responses. However, our understanding of how different vaccine types elicit durable antibody responses is lacking. To assess the impact of vaccine type on antibody responses, we measured IgG isotypes against four consensus HIV antigens from 2 weeks to 10 years post HIV-1 vaccination and used mixed effects models to estimate half-life of responses in four human clinical trials. Compared to protein-boosted regimens, half-lives of gp120-specific antibodies were longer but peak magnitudes were lower in Modified Vaccinia Ankara (MVA)-boosted regimens. Furthermore, gp120-specific B cell transcriptomics from MVA-boosted and protein-boosted vaccines revealed a distinct signature at a peak (2 weeks after last vaccination) including CD19, CD40, and FCRL2-5 activation along with increased B cell receptor signaling. Additional analysis revealed contributions of RIG-I-like receptor pathway and genes such as SMAD5 and IL-32 to antibody durability. Thus, this study provides novel insights into vaccine induced antibody durability and B-cell receptor signaling.

## Introduction

While vaccine-elicited antibody durability has been achieved for licensed vaccines such as yellow fever, measles, smallpox, and Hepatitis B, elicitation of long-lived, functional antibody responses with candidate HIV-1 vaccine regimens remains elusive^1,2^. In the case of experimental HIV vaccines, efficacy is correlated with Envelope (Env)-specific antibody responses^3,4^. In particular, Env IgG3 titers that mediate antibody-dependent cellular phagocytosis (ADCP) and antibody dependent cellullar cytotoxicity (ADCC) are correlated with decreased risk of infection^5,6^. However, these responses decay more than 10-fold 6-months post-vaccination, paralleling the decline in vaccine efficacy^7^. Estimates of HIV-1 specific IgG half-lives range from 25-213 days, depending on the vaccine regimen and antigen (excluding gp41)^7–9^. Interestingly, the Vaccine-Induced HIV Seropositivity/Reactivity (VISP/VISR) in non-infected HIV vaccine recipients is commonly observed for vaccines with a Gag or Env component^10^, indicating that antibody responses can remain detectable for several weeks to a year post-vaccination. However, positive results on diagnostic tests are qualitative in nature and thus not quantitative in estimating antibody longevity. Hence, in this study, we measured antibody responses in the VISP cohort by a quantitative assay, the Binding Antibody Multiplex Assay (BAMA), across vaccine regimens. Furthermore, we model antigen-specific antibody decay kinetics and immunoglobulin subclasses necessary to inform rational design of HIV-1 vaccines with improved durability.

Vaccine induced antibodies are secreted by plasmablasts within days of exposure, followed by memory B cells and plasma cells. These B cell dynamics are noticeable in analyses of half-lives using antibody-titers^11,12^. Env-specific antibodies after gp120 protein vaccination correlate with the number of circulating short-lived memory B-cells^13^; however, in participants who control HIV without treatment, memory B cell responses did not correlate with plasma antibody titers^14^. Since memory B cells differentiate into plasma cells upon boost and studying the plasma cell compartment in human participants is difficult, memory B cells provide the best window into signaling underlying the generation of antibody responses.

**Table 1:** Table of vaccination strategies for trials studied.

Trial arm	Strategy	Products	Envelope	Month							
				0	1	2	3	4	6	8	10
097-T1	CCPP	vCP1521, AIDSVAX	gp120	C	C		C + P		C + P		
097-T2	CCPP	vCP1521, AIDSVAX	gp120	C	C		C + P		C + P		
105-T2	DDPP	HIV-PT123, AIDSVAX	gp140, gp120	D	D		P		P		
105-T3	DDD/P	HIV-PT123, AIDSVAX	gp140, gp120	D	D		D + P		D + P		
105-T4	D+P	HIV-PT123, AIDSVAX	gp140, gp120	D + P	D + P		D + P		D + P		
094-T1	DDMM	GEO-D03, MVA/HIV62B	gp160, gp150	D		D		M	M	M	
094-T2	DDMMM	GEO-D03, MVA/HIV62B	gp160, gp150	D		D		M	M		M
094-T3	DDMM	GEO-D03, MVA/HIV62B	gp160, gp150	D		D		M		M	
205-T1+3	DDMM	JS7, MVA/HIV62	gp160, gp150		D		D		M	M	
205-T4	MMM	MVA/HIV62	gp160, gp150	M		M			M		

Main trials considered in this study (additional trials are in Supplementary information). Envelope gives which component of the envelope was included in the trial vaccines. Blank indicates no injection given at month. All protein vaccines here use AIDSVAX B/E. C indicates Canarypox vector, P indicates protein, D indicates DNA, rAd represents recombinant adenovirus and M represents Modified vaccinia ankara.

Additional insights into drivers of vaccine induced antibody responses from genome-wide transcriptional profiling have revealed gene signatures of B cell differentiation and early innate signaling. Early studies of gene expression showed type I interferon, inflamasome, and complement signaling in peripheral blood mononuclear cells (PBMCs) upon vaccination^15–24^. Specifically, analysis of early innate PBMC signatures associated with antibody levels after one dose of recombinant HIV gp140 with TLR-4 agonist adjuvant revealed a negative correlation of mitotic cell division modules with antibody production and upregulation of plasma cell surface markers a week after vaccination^23^. Further, a recent study identified a B cell (CD14^−^CD20^+^) gene signature that predicts protection from infection after vaccination with Ad26-prime and gp140-boost in Rhesus Macaques and in RV-144^24^. Collectively, these results suggest that gene expression profiling, particularly from sorted B cells may unveil important insights into mechanisms of B cell differentiation across vaccine types.

In the present study, we assessed the longevity of HIV-1 Env-specific IgG and IgG3 responses across 10 HIV vaccine regimens and quantified the effects of each vaccine type on durability using both linear and nonlinear mixed effects modeling (see Table 1). Genome-wide transcriptional profiling of antigen-specific B cells in MVA and protein-boosted trials was also performed to understand the B cell associated pathways underlying durability in participants. Thus, this study performs cross-protocol analysis revealing an association between HIV vaccine type and both B cell gene expression and antibody durability. Interestingly, analysis of antibody half-lives suggest elicitation of previously described long-lived plasma cell responses upon HIV vaccination.

## Results

### Summary of data and vaccine regimens

Five phase I or phase IIa HVTN trials 077 (DNA/Ad35 or Ad5), 094 (DNA/ HIV62B MVA), 097 (ALVAC/AIDSVAX B/E), 105 (DNA/AIDSVAX B/E), 205 (DNA/HIV62B MVA) were investigated in this study. Broadly, HVTN 094 and HVTN 205 were MVA-boosted trials with or without DNA priming, utilizing DNA expressing VLPs displaying gp160 in the prime and matched MVA-expressing VLPs displaying gp150 in the boost, HVTN 077 was Adenovirus (Ad)-boosted, and HVTN 097 and HVTN 105 were protein-boosted with subtype B MN gp120 and subtype A/E A244 gp120. HVTN 097 was primed with canarypox containing clade E 92TH023 gp120 while 105 was primed with DNA containing clade C ZM96 gp140. HVTN 094-T3 and 105-T2 were each primed twice with DNA followed by two MVA or protein boosts, respectively (Table 1), thereby enabling a comparison between DNA-MVA and DNA-protein trials. In addition, samples from the HVTN 910 cohort exhibiting [Vaccine Induced SeroPositivity (VISP) or Vaccine Induced SeroReactivity (VISR)] were analyzed up to 10 years post-last vaccination^10^. IgG or IgG3 in serum were assessed by a Binding Antibody Multiplex Assay (BAMA) for binding to a panel of consensus HIV Env proteins including gp120, gp140, gp41, and the V1V2 region of gp120 at peak (2-4 weeks post-last vaccination) at multiple post-vaccination time points. Vaccine immunogens varied by trial with DNA-MVA (HVTN 094 and 205) utilizing a gp150 immunogen, and Canarypox-protein (HVTN 097) and DNA-protein (HVTN 105) utilizing a gp120 immunogen.

**Figure 1:**
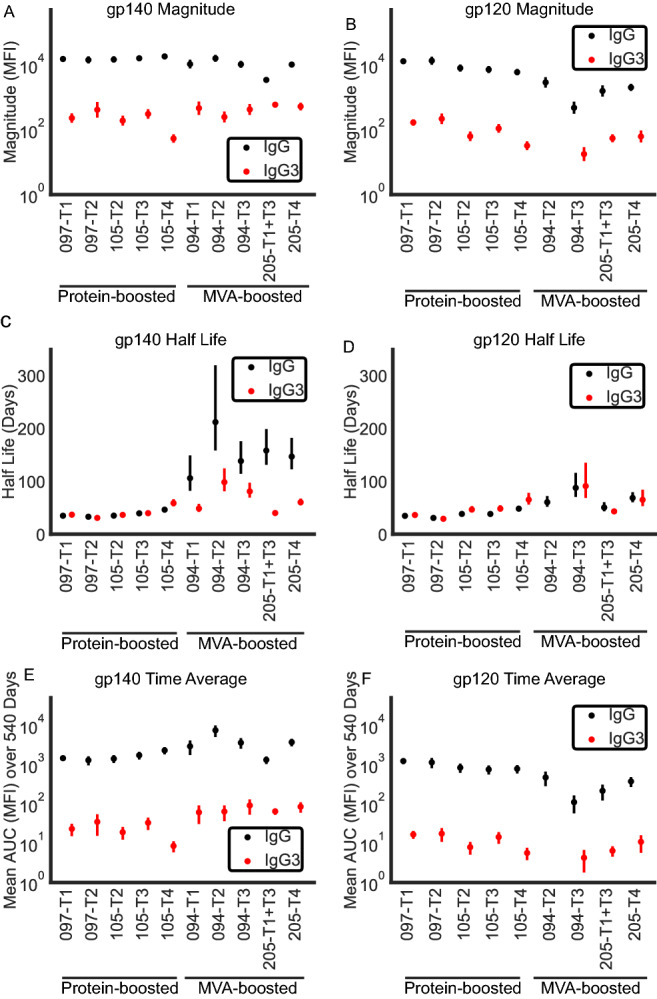
Results of linear model: peak magnitudes (mean MFI for trial across individuals), half lives, and time-averaged mean response of IgG and IgG3 responses to Con S gp140 and Con 6 gp120. Estimated magnitude of response at peak (net MFI) to (**A**) gp140 and (**B**) gp120. Estimated half life of antibody response (days) to (**C**) gp140 and (**D**) gp120. Estimated time-averaged mean response to (**E**) gp140 and (**F**) gp120 across first 540 days after first vaccination. Error bars represent standard error. Refer to Table 1 for the trial-specific strategies. Color represents the isotype of antibody (black IgG, red IgG3). 105-T3 (DNA-protein) lacked sufficient nonzero IgG3 responses against gp120 in the first 540 days post-peak for inclusion.

**Figure 2:**
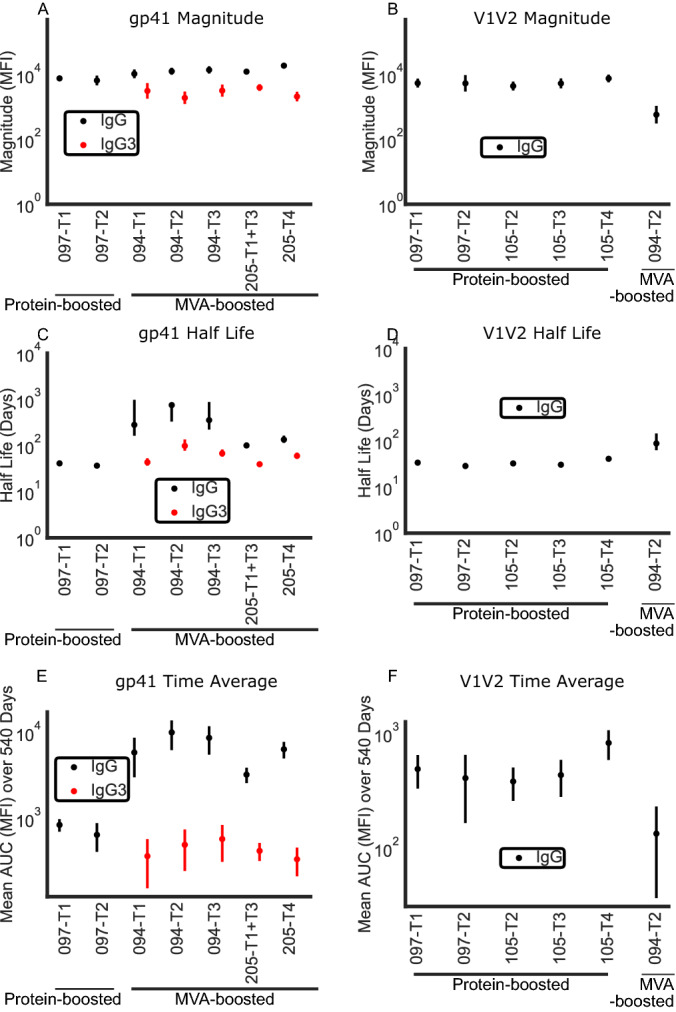
Results of linear model: peak magnitudes (mean MFI for trial across individuals), half lives, and time-averaged mean response of IgG and IgG3 responses to gp41 and gp70_B.CaseA_V1V2. Estimated magnitude of response at peak (net MFI) to (**A**) gp41 and (**B**) V1V2 region. Estimated half life of antibody response (days) to (**C**) gp41 and (**D**) V1V2 region. Estimated time-averaged mean response to (**E**) gp41 and (**F**) V1V2 region across first 540 days after first vaccination. Error bars represent standard error. Refer to Table 1 for the trial specific strategies. Color represents the isotype of antibody (black IgG and red IgG3). Many trials lacked sufficient response to V1V2 (IgG and IgG3) and gp41 IgG for inclusion in model. 097-T1 data for gp41 IgG3 was excluded based on lack of decrease from peak to subsequent time point.

8380 individual measurements were collected from 557 participants. Antibody responses varied greatly among vaccine trials and by epitope specificity. gp140 responses were the largest and most frequent responses, followed by gp41, gp120, and V1V2 responses (Figs. 1, 2, Table S1). Furthermore, Env specific IgG persisted > 6 years (2532 days (d), Figs. S1, S2). In contrast, gp120-specific IgG3 were short-lived and only present up to 6 m (174 day) post-vaccination. In the current study, data from 325 participants was included in linear models based on the following criteria: non-zero mean fluorescent intensity (MFI) [defined in Methods] at peak immunogenicity, MFI was 3x greater than the subject-specific baseline at the peak time point, MFI was greater than the subject-specific baseline at a second (nonzero) post-peak time point. Since the antibody levels are expected to decay over time, only trials in which Net MFI decreased between peak and subsequent time point were considered. No arms Adenovirus strategies (HVTN 077) met these criteria for linear modeling due to a paucity of data in the first 540 days but sufficient amount of data from VISP cohort. Hence, these were analyzed in the supplement and additional information is available in Fig. S3.

### Durable antibody responses in MVA-boosted regimens

To investigate antibody half lives across protocols, linear mixed effects model was developed using trial arm as fixed effects and participant ID as random effect. Further, time-averaged mean antibody responses were calculated to summarize average antibody response across the entire 540 day (1.5 years) window of the model.

Robust responses against gp140 were elicited in both MVA (HVTN 094, 205) and protein-boosted trials (HVTN 097, 105). For gp140 and gp120 IgG as well as gp140 IgG3 antibody responses, MVA-boosted trials (HVTN 094, 205) displayed longer half lives than protein-boosted trials (HVTN 097, 105) (p < 0.005 for each comparison of 105-T2 and 094-T3, the most closely matched regimens, Fig 1). These results were consistent across all MVA trials. IgG3 displayed similar half lives but peak magnitudes were 1.13-5.83 natural logs lower than IgG responses across antigens. Peak magnitudes varied among antigens with gp140 > gp41 > gp120 > V1V2 (Figs. 1, 2). gp41 responses, as expected, were not present in these protein-boosted regimens (since they had gp120 boost). IgG responses for MVA-boosted regimens against gp41 were robust both in peak magnitude and in a prolonged half life (Fig. 2). MVA-boosted trials had strikingly attenuated responses against gp120, despite having gp140 IgG magnitudes similar to protein-boosted trials (Fig. 1). Attenuated peak magnitude resulted in lower time-averaged antibody responses against gp120 in DNA-MVA regimens (HVTN 094, 205) than DNA-protein regimens (HVTN 097, 105), despite exhibiting a longer half-life (836 vs 111 MFI for 105-T2 vs 094-T3, the two most closely matched trials, Fig. 1, Table S1). Note that demographic variables were not included in this analysis due to limited sample sizes required for mixed-effects modeling. Fig S4 shows that despite demographic differences between trials, there were no demographic variables that were systematically different in all MVA trials or all protein trials, indicating that these differences do not confound the main conclusions of our study.

Antibody responses to V1V2 were fewer and of lower magnitude than those against other antigens, and IgG3 responses were particularly rare (Fig. 2). As with gp120, protein-boosted trials (HVTN 097, 105) had higher peak magnitudes of V1V2 IgG than MVA-boosted trials (HVTN 094, 205), although this difference was not statistically significant. DNA/protein (co-administered 4 times, 105-T4) had the largest time-averaged IgG response to V1V2 (831 MFI) with canarypox-canarypox/protein (492 MFI, 097-T1) exhibiting the next highest time-averaged response. No trial arms had sufficient IgG3 responses to V1V2 for statistical modeling. Thus, trends in our data suggest that DNA/protein administration as in 105-T4 may produce a larger IgG response against V1V2, but this needs to be confirmed with larger scale study (statistically non-significant).

### Analysis of persistent antibody responses

**Figure 3:**
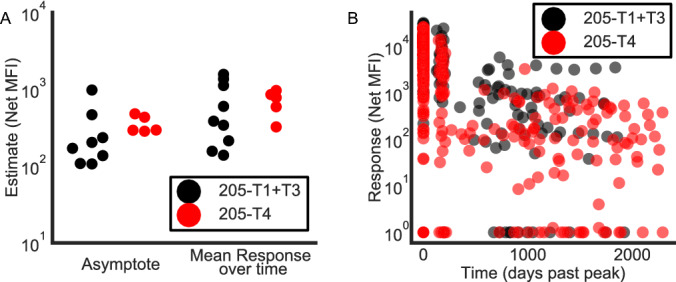
Antibody responses can persist at high levels for up to a decade. (**A**) Estimated asymptote and time-average mean antibody response are shown for IgG responses to gp140. Only regimens with participants who still had levels of that antibody $>100$ Net MFI after 180 days are included. (**B**) IgG response levels against gp140 shown for all individuals in HVTN 205 with day 0 representing peak response at 2 weeks after vaccination.

To further probe long-term responses that persist up to 10 years post-vaccination, a nonlinear mixed effects model was constructed for gp120 and gp140 to estimate the asymptote in the antibody-decay curve. The durable responders whose last two measurements were within 100 MFI of each other and had a nonzero average were included in this analysis and stable minimums were modeled as asymptotes. Durable responders were defined as > 100 and 3x (pre-vaccination) baseline Net MFI at peak and 6 month timepoints. Post vaccination data points that were less than (pre-vaccination) baseline were not included. Only viral-boosted trials HVTN 077 (Ad5), HVTN 094 (MVA), and HVTN 205 (MVA) had sufficient post-vaccination data to be modeled. Newer trials (HVTN 097, 105) have not yet sampled for a sufficient period post-vaccination to observe long-lived responses, and other trials did not have sufficient participants with maintained antibody responses. DNA/MVA vaccination in HVTN 205 resulted in an asymptote between $10^2$ to $10^3$ (Fig. 3) for gp140 (mean = 356 MFI). This long-lasting response was recapitulated in an adenovirus-vectored trial (HVTN 077, mean = 413 MFI, Fig. S3). Uniquely, all participants modeled in 077 had time-averaged mean response > 103 Net MFI as also indicated by a late follow up past first 540 days (Fig. S3), indicating that Ad26 vector, in addition to MVA, can generate long-lasting antibody responses to HIV vaccination. Baseline (pre-vaccination) measurements made a negligible contribution in all cases (Net MFI $< 5$). Interestingly, gp120 mean asymptote had a tight range (Fig. S2) for MVA boosted regimens and a comparable time-averaged mean response (HVTN 094-T2, T3, HVTN 205 T4). The similarity between asymptotic responses to MVA vaccination alone (205-T4) and DNA prime/MVA boost (205-T1 + T3) can be seen clearly in response to gp120 (Fig. S2, panel D). Taken together, these data indicate that viral boosted trials can induce lasting, stable, quantifiable, long-term responses in a subset of participants.

### Differential activation of B cell molecular response by MVA and protein-boosted trials

**Figure 4:**
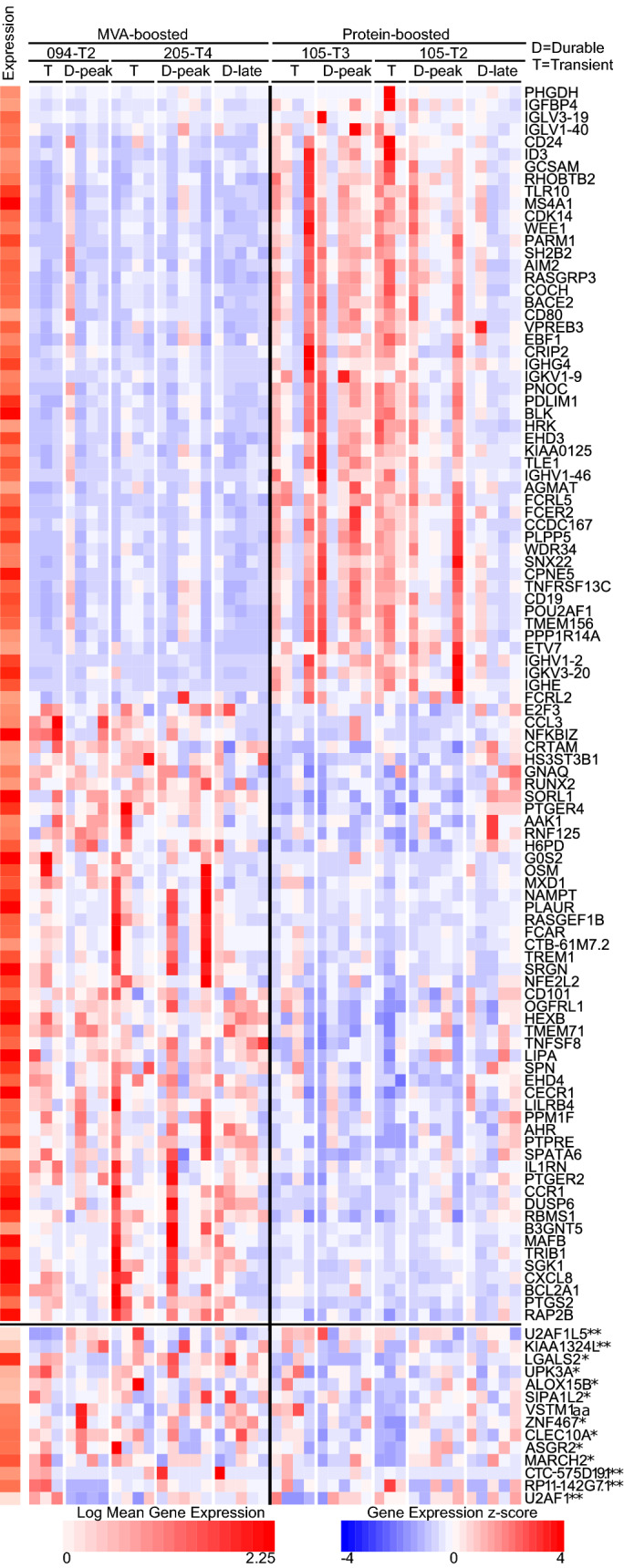
Transcriptional signatures of MVA vs Protein boost and durability in B cells. Heatmap of normalized expression (z-score) with (far left) mean expression over all samples. Top heatmap (above the horizontal black line) of differentially expressed genes (with p-adj $< 0.005$, 50 largest log fold change) in each direction for MVA vs protein comparison. Bottom heatmap (below the horizontal black line) of genes differentially expressed (p-adj $< 0.05$ and log fold change (LFC) $> 0.5$ ) between durable vs transient participants (*105-T2 and **094-T2) at peak in at least one trial and with no change (LFC $< 0.5$) between durable peak and late timepoints in either trial. Labels at top indicate sample groups with a black line between MVA and protein-boosted groups. D- durable and T-transient. Peak and late (6 months post-peak) time points are shown for durable participants.

We next investigated molecular signatures of durable responses in $CD19+CD20+IgD-CD27+ConS gp120+$ B cells obtained from durable responders at peak and 6 months post-last vaccination. B-cells were flow sorted and subjected to RNA sequencing. Transient responders at peak immunogenicity timepoints were also analyzed for comparison. Durable responders were defined as $> 100$ and 3x (pre-vaccination) baseline Net MFI at peak and 6 month timepoints while transient responders were $> 100$ Net MFI at peak but not six months. HVTN 094-T2 (DDMMM), 105-T2 (DDPP), 105-T3 (DDD/P), and 205-T4 (MMM) vaccine regimens were selected due to the high percentage of participants with durable gp120 IgG responses and sample availability. First, responses to MVA-boosted (HVTN 094, 205) and protein-boosted (HVTN 105) regimens at peak were directly compared to assess molecular mechanisms driving differences in observed antibody responses. 309 and 439 genes exhibited significantly elevated expression in MVA-boosted and protein-boosted regimens respectively, after adjusting for BMI and gender (p-adj $< 0.05$ and LFC $> |0.5|$). Protein-boosted trials (HVTN 105) induced several markers of B cell function and lineage including CD19, CD40, and variable region immunoglobulin genes (Fig. 4 and Additional data table S1). Several immune signaling genes including TNFRSF13C, TLR10, FCRL5, FCRL2, FCRL1, and FCRL3 were elevated in protein-boosted trials. Pathway analysis of these genes suggested activation of B cell receptor signalling, B cell activation, and B cell proliferation (p-adj $< 0.05$, Additional Data Table S2) leading to a higher magnitude of antibody responses in protein-boosted trials. However, MVA-boosted trials (HVTN 094, 205) led to increased expression of important immune regulators such as RUNX1, RUNX2, and TLR4 (Fig. 4 and Additional Data Table S1). Furthermore, pathway analysis revealed increased activity of Lysosome, Toll-like Receptor Signaling, Granulocyte, and IL-1R pathways in MVA-boosted trials (p-adj $< 0.05$, Additional Data Table S2). Taken together, the results suggest attenuation of critical B cell signaling in MVA-boosted trials (HVTN 094, 205) and an interesting signature of FCRL genes in protein-boosted trials (HVTN 105).

**Figure 5:**
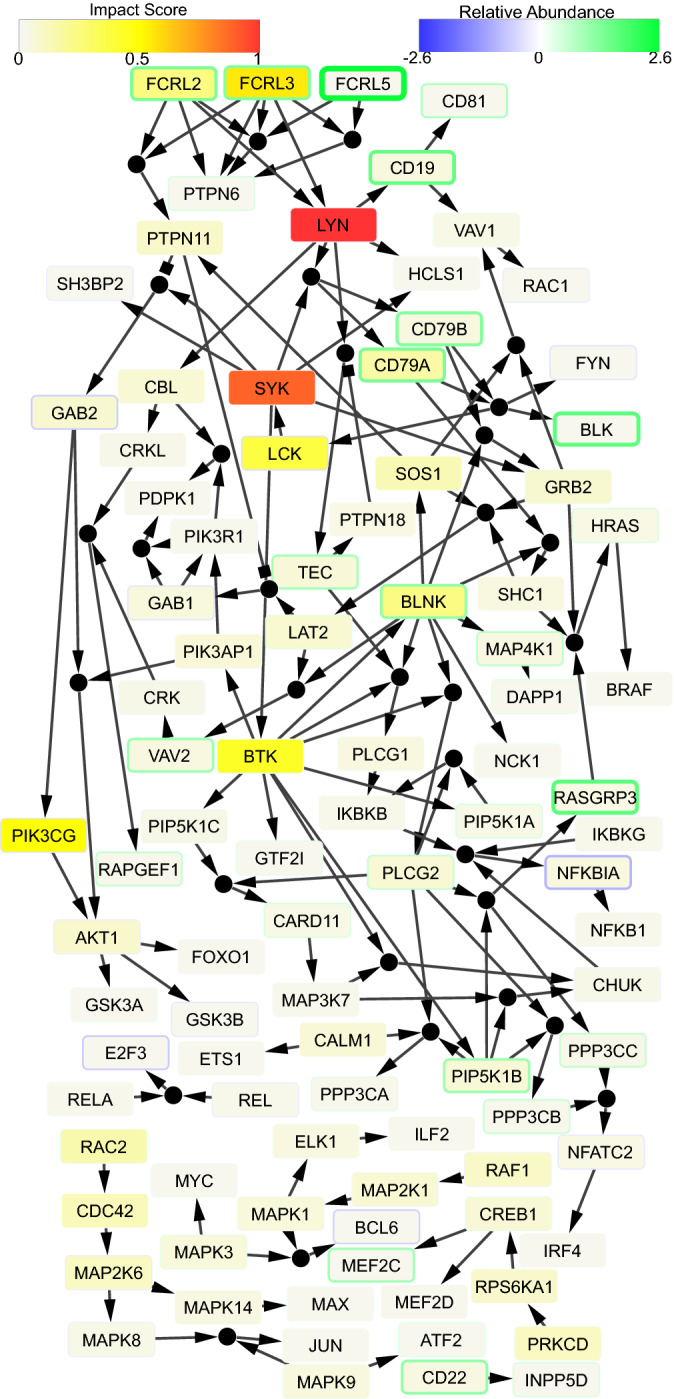
Regulation of BCR Signaling Pathway with FCRL genes. The network depicts differential regulation of BCR signaling pathway with FCRL genes. Small circular nodes indicate joint/synergistic regulation of downstream genes (‘and’ rules) whereas multiple incoming edges to a rectangular node indicate that anyone of the upstream nodes can regulate downstream nodes (‘or’ rule). Colors of the rectangles ranging from white to red indicate low to high impact score. Widths of the rectangles’ outlines and their color ranging from blue to green indicate fold difference (mRNAs) between protein-boosted and MVA-boosted participants at peak. Blue represents higher expression after MVA boost and green represent higher expression in protein boost. Impact scores have been divided by the largest impact score in the pathway.

Discrete dynamic modeling was employed to better understand changes in BCR signaling and how they relate to changes in FCRL genes. A network of BCR signaling (NetSlim and Wikipathways^25,26^) was used with and without addition of FCRL genes to evaluate the role of FCRL genes in the regulation of BCR signaling. The FCRL genes were incorporated in BCR signaling pathways by mining primary literature^27,28^. Boolean Omics Network Invariant-Time Analysis (BONITA), which performs discrete-state modeling identified BCR pathway with FCRL genes to be significant at p $<0.001$^29^ between MVA and protein boosted groups. PTPN6 and PTPN11 inhibit BCR signaling and were identified to have low impact on regulation of BCR pathway by BONITA irrespective of FCRL genes. The regulation identified by BONITA suggests that FCRL2 and FCRL3, which have very high impact scores, are required for activation of PTPN11 and PTPN6 while LYN can be activated by either Fig. 5. Interestingly, BONITA revealed that both CD79A and CD79B components of BCR are required for downstream signaling. Thus, we can conclude that continued low impact of PTPN6 and PTPN11 paired with high impact of FCRL and LYN indicates that FCRL molecules act through LYN to activate BCR signaling. The rule-based learning suggests an activating role of FCRL molecules following protein boost.

### B cell signature of durable response

We next investigated differences in the gene-expression of participants with durable responses (defined by $>100$ Net MFI and 3x (pre-vaccination) baseline measured at least 180 days after peak) vs. transient responders (comparison of durable vs transient responders at the peak) within each trial arm. In order to identify genes with persistent differences across time, we identified genes which were differentially expressed between trasient and durable responders in at least one trial and did not change from peak to six months in both protein-boosted (105-T2) and MVA-boosted (205-T4) trials. There were 14 such genes, 9 were differentially expressed in protein-boosted (105-T2) and 5 were differentially expressed in MVA-boosted regimens (094-T2). Many of these genes have immunomodulatory functions, including LGALS which was differentially expressed in both 105-T2 and 094-T2 and binds to lymphotoxin alpha, a potent immunomodulator. Other genes included VSTM1 which interacts with Fc receptors and CLEC10A which is expressed in highly active thymic B cells^30^. We further sought to understand molecular pathways underlying changes in gene expression between durable and transient responders using BONITA software^29^. The RIG-I-like receptor and Cytosolic DNA sensing pathways were significantly different and were highly expressed in transient vs. durable vaccine responders at peak in MVA-boosted HVTN 094-T2 ( p $< 0.01$, Additional Data Table S3). However, no significant pathways were observed in protein-boosted 105-T2 (p$>0.05$). Thus, durable responses were driven by underlying transcriptional changes within a same trial and might be regulated differently in MVA- and protein-boosted HIV vaccination. However, more sample size and B cell subtypes should be studied in future to address understand transcriptional regulation of durable responses.

### Gene-expression changes are associated with antibody half-life

**Figure 6:**
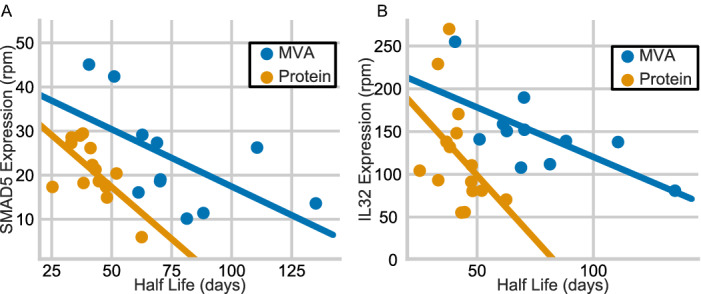
Genes associated with half life. Scatter plot depicting association (p $<0.001$ and |*r*| $> 0.7$) between gp120 IgG half life and (**A**) SMAD5 and (**B**) IL32 expression at peak. Color represents Protein-boost vs MVA-boost. Complete list of correlated genes is shown in Additional data table S4. Line shown on graph is from regression model fit within Seaborn^41^.

An association of B cell gene expression at peak antibody magnitude with antibody half life suggests potential molecular mechanisms responsible for long-lived antibody responses. This association could be dependent on the vaccine type used in each trial, and thus MVA- and protein-boosted trials were analyzed separately (see methods). Twenty-four genes were significantly associated with half-life in MVA (HVTN 094, 205) and in protein-boosted trials (HVTN 105) (Additional data table S4). These genes included tetraspanins (SNX13 and TSPAN33) which are involved in several signal transduction events required for B cell activation^31,32^ and ubiquitin-ligases (UBE3C, HERC3, and RAB40C), in agreement with previous vaccine studies^18,33^. IL32, shown to act as an immunosuppressant during HIV infection^34^, was accordingly identified as negatively correlated in both MVA- and protein-boosted trials (Fig 6). Additionally, SMAD5, a key TGF-$\upbeta$ signaling gene^35^, was also found to be negatively associated with half-life (Fig. 6), in consensus with its role in transducing signal from BMPs (Bone Morphogenic Proteins) that suppress B cell responses^36^. Of the 24 correlated genes, 6 are targets of TCF3, NFYB, and ZMIZ1 transcription factors^37,38^. TCF3 is an important factor for development of B cells and differentiation into plasma cells^39^. Interestingly, the transcription factor NFY is known to regulate interferon-γ modulated Major Histocompatibility Complex class II (MHC-II) genes in B cells^40^. Taken together, this analysis shows that the durability of antibody responses is induced by a coherent transcriptional signature supported by previous studies of B cell gene expression.

### Comparison to B cell signatures from previous vaccine studies

To compare gene signatures identified in this study with previous vaccine signatures we investigated influenza and diphtheria toxoid vaccine response. Comparison between live attenuated (LAIV) or protein (TIV) influenza vaccination revealed that LAIV produced lower magnitudes of HA-specific antibodies and gene expression at 1 week than TIV^18^. Genes highly expressed after MVA-boosted vaccination (HVNT 094, 205) were enriched in genes downregulated after LAIV compared to pre-vaccination. Further, genes highly expressed in durable vs transient subjects in protein-boosted 105-T2 (DDPP) were also enriched for genes downregulated after LAIV. Further, a signature of many immunoglobulin genes, TNFSF13B, and FCRL5 was observed seven days after vaccination with TIV or diphtheria toxoid^22^ as well as in protein-boosted HIV vaccines in our study. Thus, protein-boosted vaccination induces genes regulating BCR signaling.

A recent study^24^ derived two gene signatures of HIV vaccine efficacy, one of 53 genes and the other of 200 genes from B cells following Ad26-gp140 vaccination in non-human primates. These signatures were validated in PBMCs from RV-144. Both signatures were enriched in genes with higher expression upon MVA boost than protein boost (p $< 10^-6$ for both). Interestingly, in protein-boosted trial 105-T2 (DDPP) the 200 gene signature was enriched in genes with elevated expression in subjects with durable response than transient response (p $< 0.005$). Thus, all responses to MVA-boosted vaccination but only durable responses to protein-boosted vaccination, induced a signature associated with protection from acquisition of HIV in human and non-human primate vaccine trials.

## Discussion

This study describes differences in half life of antibody response between MVA-boosted and protein-boosted HIV vaccinations. MVA-boosted vaccinations (HVTN 094, 205) lead to longer half life with lower-magnitude of antibody responses than protein-boosted HIV vaccines (HVTN 097, 105). Nevertheless, protein-boosted vaccines elicited a greater time-averaged mean antibody response to gp120. Promisingly, MVA-boosted vaccines led to half lives longer than those for immunoglobulin molecules^42,43^ and short-lived plasma cells^44^, but orders of magnitude shorter than responses driven by long-lived bone marrow-resident plasma cells^12,45^. In particular, gp120-specific antibody response kinetics were comparable to previously observed short-lived memory B cell responses^12^. Moreover, memory B cells produce the primary, short-lived, Env-specific antibody response to HIV infection and protein-only vaccination^13^. The half-lives in our data suggests that MVA-boost may induce a similar memory B cell-dependent response while protein-boost induces a short-lived plasma cell response. Nonetheless, the recognizable asymptote observed in this study in viral-boosted trials indicates that long-lived plasma cell responses are possible. Achieving an efficacious HIV vaccine may require identifying a vaccine strategy, including immunogen and method of protein delivery (e.g. vector or adjuvant^46^), which results in responses of the size of those in protein-boosted trials but with extended half lives in multiple years and decades.

Antibody responses observed here and in primary trials vary considerably by antigen-specificity. We found responses to gp140 are most frequent and robust followed by gp41 (when included in boost) and gp120 with few responses to V1V2^47–53^. When gp41 is included in the boost (i.e. gp150 boost vs gp120 boost), gp41 responses were as robust as gp140 responses, with increased response longevity vs gp120 responses. High epitope similarity to gut bacteria may contribute to rapid, large gp41 responses and persistence of gp41-specific antibodies in early HIV infection and HIV vaccination^54,55^. Inconclusivity of this study with regard to V1V2 along with putative efficacy of V2-specific antibodies calls for a higher powered study of V2-specific antibody responses^3,56,57^, including ongoing efficacy trials.

In the current study, effects of immunogen on vaccine-elicited antibody durability was not explicitly tested because MVA and protein boosted regimens used gp150 and gp120 respectively. Moreover, protein-boosted regimens (HVTN 097 and HVTN 105) included in this study were boosted with AIDSVAX B/E (gp120 immunogen) whereas MVA-boosted regimens (HVTN 094 and HVTN 205) analyzed in this study were boosted with MVA/HIV62 (gp150 immunogen) matched to the DNA prime. Despite the differences in gp120 sequences previous studies suggest a stronger response regardless of the immunogen used. Moreover, viral-prime, protein-boost vaccine trials have demonstrated robust gp120-specific antibody response following both gp120 and gp140 boost^58–60^. Taken together, this evidence demonstrates that vector and priming strategies, independent of immunogen, play a critical role in determining the magnitude of the gp120-specific antibody response.

The nonlinear model demonstrated that two different viral vectors (vaccinia and adenovirus) can lead to long-lasting antibody responses. Nonetheless, $\sim 100x$ difference between the estimates of initial magnitudes and asymptotes indicates that few of the antibody producing cells generated at peak survive as long-lived plasma cells in individuals with persistent responses. Interestingly, the level of persistent response correlated with the initial response (Spearman r=0.55 for the asymptote and magnitude, p = 0.018 for gp140). This implies that increasing the immunogenicity of the boost could increase the asymptote levels as well as suggested by the Imprinted Lifespan model of plasma cell survival^61^. Despite the caveats of the HVTN 910 (VISP/VISR) cohort (positive responders only, subset of original trials), we have added further evidence that long-lived vaccine-induced antibody responses are achievable with current HIV vaccine regimens. Moreover, this evidence makes clear that future experimental vaccines studies should investigate persistence of antibody responses and underlying B cell transcriptional programs.

Our study profiled gene expression in memory B cells, which develop upon multiple rounds of vaccinations and whose predecessors, at the minimum, would have contributed to the development of long-lived plasma cells. Although plasma cells develop distinct transcriptional programs from memory B cells^62^, their lifespan is likely imprinted at the time of differentiation^12^. Long term survival of antibodies has been shown to correlate to memory B cell numbers following meningitis vaccine^63^. Moreover, gp120-specific antibodies correspond to memory B cells following HIV vaccine^13^. Therefore, study of gene expression in memory B cells is an attempt to understand responses that might have led to lasting memory B cell and plasma cell responses. Though our study can not differentiate into responses driven by specific B cell types, it demonstrates the state of memory B cells that emerge from germinal centers at the same time as longer-lasting MVA-induced plasma cells. Given the difficulty of sampling lymph nodes in human participants and pre-selection of participants, memory B cell signaling provides the most expedient window into germinal center dynamics.

The current gene expression analysis indicated a clear and strong signature of difference between protein and MVA boosted trials including differences in FCRL genes. FCRL molecules have an excitatory role in marginal zone (B2) B cell signalling^27, 64–67^. Markedly high FCRL2, FCRL3, and FCRL5 were identified in the context of Hepatitis C vaccination^68^. T-bet and FCRL5+ memory B cells correlate with longevity of antigen-specific antibody responses^69^, however there are conflicting data on longevity and FCRL5+ memory B cells^70,71^. We found a weak negative correlation between Tbet and half life in MVA trials (HVTN 094, 205) and no other correlations. Finally, discrete state modeling shows that FCRL2 and FCRL3 can independently activate LYN which leads to downstream activation of the BCR signaling pathway^27^. Thus, the current study provides mechanistic insights into regulation of BCR signaling by FCRL.

The transcriptomic signature of enhanced durability of MVA-boosted regimens (HVTN 094, 205) consisted of RUNX1, RUNX2, and TLR4 as well as higher levels of Toll-like Receptor and IL-1R signaling. Thus, use of TLR4 agonists could increase antibody durability. Indeed there are safe TLR4 adjuvants that have already been tested in HIV vaccination^72–74^. Unfortunately, dendritic cell-based therapeutic HIV vaccination studies demonstrated decreased immunogenicity with TLR4 activation^75^. Another adjuvant, MF59, used in HVTN 702 (NCT02968849) with gp120-boost following canarypox prime, increases strength, breadth, and durability of response to Influenza vaccine by activating the early innate response^76^. Thus, the data shown here calls for careful understanding of how TLR4 agonists affect antibody responses on their own and in the context of MF59.

Enrichment of HIV vaccine signatures associated with protection^24^ in MVA-boosted trials along with increased gp120 half-lives is encouraging for the development of MVA as a vector for HIV vaccination. Moreover, enrichment of this signature in subjects with durable responses in AIDSVAX B/E protein-boosted 105-T2 suggests that protective HIV vaccine signatures may also be associated with durable responses. The Ehrenberg et al. gene signatures was identified by measuring gene expression at varied time points in human PBMCs (RV144) and in B cells from non-human primate Ad26-gp140 efficacy studies^24^. However, there are many differences between our study and Ehrenberg et al^24^, including antigen specificity (gp120 vs all B cells), timepoints assessed (2 weeks and 6 months vs multiple), and prime (DNA vs Ad26 or canarypox). Nonetheless, MVA-boosted regimens (HVTN 094, 205) produced a B cell gene signature associated with protection, but at lower levels of antibody production than the protein-boosted regimens in Ehrenberg et al. Thus, our data present a rationale for continued investigation of MVA+ protein regimens, including testing immunogen to improve magnitude and durability of antibody responses.

## Methods

### Measurement of serum Ab levels

Antibody levels measured by binding antibody multiplex assay (BAMA) were either acquired from the primary analysis or obtained de novo for the current study. We tested serum samples from HVTN trial 910 retrospectively with IRB approval (Rochester: RSRB00065290, Duke: #Pro00077889). Longitudinal samples from HVTN 105 at month 12 were obtained with informed consent (Rochester: RSRB00065290, Duke: #Pro00077889). Primary data for trials HVTN 077^47^, HVTN 094^48^, HVTN 097^49^, HVTN 105^50^, HVTN 205^53^ were previously acquired and data were used in this analysis with approval from the HVTN^77^. Analysis of Levey-Jennings charts indicate the comparability of historical controls across trials and time for positive controls including HIVIG and CH58 mAb. All methods were carried out in accordance with relevant guidelines and regulations, and informed consent was obtained from all subjects.

Serum samples were tested for IgG and IgG3 binding Ab responses to gp41, Con S gp140^78–80^, Con6 gp120, and gp70_B.CaseA_V1V2 at primary and longitudinal timepoints as described previously^3,54,81,82^. Briefly, samples were incubated with antigen-coated beads in BAMA assay diluent (5% Normal Goat serum (v/v), 1% milk blotto (w/v), 0.05% Tween-20 (v/v) in PBS), washed, then incubated with either biotinylated anti-human IgG (Southern Biotech) or biotinylated anti-human IgG3 (Calbiochem) followed by washing and incubation with Streptavidin-PE (BD Biosciences). Binding peak magnitude (Median Fluorescence Intensity) was measured in duplicate using a Bio-Plex 200 (Bio-Rad) and averaged to obtain Mean Fluorescence Intensity (MFI). Positive controls included titrated HIV positive IgG (HIVIG), CH58 mAb (Protein Production Facility, Duke) and Purified IgG3 human myeloma protein (Sigma) coupled beads. All experiments were conducted following Good Clinical Laboratory Practice (GCLP) guidelines with tracking of antigen performance through historical Levey-Jennings control charts.

### Modeling of Ab decay

Antibody decay was assessed by both linear and nonlinear mixed effects modeling of log-transformed or raw Net MFI values from BAMA respectively. Models used random effects to account for inter-individual variability but did not control for demographic variables. Linear mixed effects models included participants with at least two non-zero ($>1$ Net MFI) points in the first 540 days after the peak (2 weeks after final vaccination) with the peak measurement 3x their (pre-vaccination) baseline and the other above the baseline. Trial arms were included if they had $>4$ included participants and Net MFI decreased between peak and subsequent time point. An independent model was constructed for post-peak IgG and IgG3 responses against each antigen which included trial arm, referred to as a “group” throughout the manuscript, as a fixed effect and participant ID as a random effect on both intercept and slope (time). A list of the number of participants included in each model and the total participants tested from each trial are listed in Table 2. Some pairs of antigens and trial arms were excluded from linear models due to non-decreasing response over time; these included gp140 IgG3 as well as gp41 IgG3 response to 097-T1. Models were fit using *lme4* and statistical properties (p-value, estimated mean, standard error) computed using *emmeans* which estimates a reference grid based on the factors in the model then averages over other factors and propagates error so that confidence intervals and p-values can be computed for the factor of concern^83,84^. Residuals were well-distributed across time (Fig S5). Covariates such as BMI and sex were not included in the model since systematic bias was not observed in these variables (see Fig S4), and insufficient sample size existed to consider covariates in our mixed effects models. Time-averaged means were obtained by dividing the area under the response curve by 540 days as ${{\text {time-average}}} = \frac{1}{540}*\int _0^{540} M*e^{t*t_c} dt = \frac{M*(e^{540*t_c}-1)}{540*t_c}$ where $t_c$ is time constant, t is time in days, and M is magnitude for each trial and propagating errors by combining partial derivatives with respect to *log*(*magnitude*) and $t_c$. Finally, for ease of interpretation, time constants were converted to half lives by $t_{1/2}=\frac{ln(2)}{t_c}$ for both linear model here and nonlinear model below.

**Table 2:** Table of number of participants included in models.

Trial	Antigen:	gp120	gp120	gp140	gp140	gp41	gp41	V1V2
	Participants tested/ Isotype:	IgG	IgG3	IgG	IgG3	IgG	IgG3	IgG
097-T1	55	48	26	48	16	35		17
097-T2	18	14	9	14	6	7		5
105-T1	25	11		11	5			
105-T2	26	18	11	18	15			17
105-T3	26	18	13	19	16			14
105-T4	25	22	13	23	21			22
094-T1	10			9	8	9	8	
094-T2	15	8		13	12	14	12	5
094-T3	13	6	5	13	12	13	13	
205-T1+T3	136	7	16	35	58	32	47	
205-T4	68	18	7	32	24	31	21	

Number of participants included in each linear model. Blank indicates that trial was not modeled (insufficient positive responses). Participants tested gives the number of participants with measured antibody responses in primary trial, except HVTN 205 which gives number of participants for which our data set contains values.

Nonlinear mixed effects modeling was performed separately for IgG against gp120 and gp140. The models were developed for durable participants (those with $>100$ MFI after 150 d ) with positive ($>1$) mean responses at the last two time points. The gp140 model included a random effect on peak magnitude and asymptote, and was developed for trials with at least 4 participants with difference $<100$ MFI and mean $>1$ for the last two time points. Further, values below (pre-vaccination) baseline were excluded. These restrictions were slightly loosened for the gp120 model because of the smaller sample size. The gp120 model included a random effect on peak magnitude and was developed for trials with at least 3 participants. The models were run using the SSasymp function and nlme package in R^85^. Time-averaged means were calculated for the nonlinear model by calculating area under the curve over ten years then dividing by time to find mean by ${{\text {time-average}}} = \frac{1}{3650}*\int _0^{3650} A+M*e^{t*t_c} dt = \frac{\frac{M}{t_c}*(e^{3650*t_c}-1)+3650*A}{3650}$.

### Measurement and analysis of B cell gene expression

Participants were selected for B cell bulk RNA-sequencing by matching durable ($>100$ Net MFI after 180 day) to transient ($<100$ Net MFI after 180 day) responders for peak Net MFI, BMI, age, gender, and geographic location as closely as possible within each group. Vials of PBMCs were requested from HVTN repository for the selected participants at peak and six month (only for participants with durable response) time points from 094-T2, 205-T4 and 105-T2. The trials were chosen for availability of 6m samples and processed by flow cytometry to isolate the gp120 specific $CD19+CD20+IgD-CD27+TetanusToxoid- Con6 gp120+$ B cells. B cells specific for Con6 gp120 were identified by staining with gp120 protein conjugated to Alexafluor 488 and Alexafluor 647, as previously described^86^. Excess anti-CD4 (Becton Dickenson) was used during cell labeling to prevent gp120 from binding T cells. On average 350 gp120+ cells were obtained per sample.

Reads were aligned and count matrices compiled for gene IDs for a total of 44 samples. Genes with read counts $<5$ in $>1/4$ of samples were filtered out. Filtered counts were converted to rpm. See Fig. S6 for quality control analysis. Differential expression (DE) between protein and MVA were identified by pooling all samples from protein-boosted and MVA-boosted trials at peak. The analysis was performed using DEseq2 with default Cook’s cutoff as well as adjustment for BMI and sex. The DE genes were selected using benjamini-hochberg adjusted q $<0.05$ and log fold change (LFC) $> 0.5$. DE analysis was also employed to compare transient to durable responders at peak in each trial independently using DESeq2 with cook’s cutoff but no adjustment for BMI or sex since these were matched between transient and durable responders within each trial. Adjusted p-value $<0.05$ and LFC $> 0.5$ were used as cutoffs. DE genes in any trial that maintained expression between peak and 6 month timepoints in both 105-T2 and 205-T4 were included in heatmap for analysis.

Genes implicated in durable response were evaluated by measuring association between gene expression at the peak and half-life estimates from linear model by Spearman correlation. The genes with coefficient of variation $>0.2$ and mean expression $>5$ within each grouping (MVA-boosted or protein-boosted) were included in this analysis. The genes with p $<0.001$ were identified for MVA and HVTN105 trials. Separately, genes correlated in both MVA and HVTN105 were identified at a less stringent p $<0.05$ in each group (MVA and HVTN105). Finally, gene-set enrichment analysis of KEGG, Biocarta and reactome gene-sets from MSigDB was performed using hypergeometric test and qusage^29,87–92^ with Benjamini-Hochberg correction. Pathway analysis of KEGG was performed using Boolean omics network invariant-time analysis (BONITA)^29,89^. BONITA was additionally used to assess the B Cell Receptor Signaling Pathway from which was taken from Wikipathways^26^ but originally created in NetSlim^25^. This pathway was manually converted into the format necessary for use with BONITA^29^. FCRL genes interact through immunoreceptor tyrosine-based activating motif (ITAM) and immunoreceptor tyrosine-based inhibitory motifs (ITIM) with LYN and PTPN molecules, respectively^27,28^. To recapitulate this behavior, activating edges were added from FCRL2-5 to LYN and activating edges were added from FCRL1-5 to PTPN6 and PTPN11. Note that PTPN6 and PTPN11 have inhibitory connections to downstream genes; activating them inhibits the rest of the pathway. The resultant networks (with and without FCRL genes) were then subjected to BONITA pathway analysis. Edge sets (.txt) and network files (.graphml) of these BCR networks are now available at https://github.com/Thakar-Lab/BONITA.

## Supplementary information

Supplementary Data Table S1.

Supplementary Data Table S2.

Supplementary Data Table S3.

Supplementary Data Table S4.

Supplementary Information.

## Data Availability

Antibody data (BAMA) and count data from RNAseq is publicly available on the HVTN Atlas portal in a folder entitled ’Palli, Seaton et al. Antibody durability’ at “https://atlas.scharp.org/cpas/project/HVTN%20Public%20Data/Cross-Protocol%20HVTN%20Manuscripts/begin.view?”.
