# Supplementary material for: Impact of vaccine type on HIV-1 vaccine elicited antibody durability and B cell gene signature
Source: Sci Rep. 2020 Aug 3;10:13031. doi: 10.1038/s41598-020-69007-w (PMC7398916; doi:10.1038/s41598-020-69007-w)
Supplement: Supplementary file 5 — Supplementary Information. [file 41598_2020_69007_MOESM5_ESM.pdf]

Supplementary Information for

Impact of vaccine type on HIV-1 vaccine elicited antibody durability and B cell gene signature

Rohith Palli, Kelly Seaton, Michael Piepenbrink, John Hural, Paul Goepfert, Fatima Laher, Susan Buchbinder,Gavin Churchyard,Glenda Gray,Harriet Robinson,Yunda Huang,Holly Janes, James Kobie, Michael Keefer, Georgia Tomaras, Juilee Thakar

Juilee Thakar.
E-mail: Juilee_Thakar@urmc.rochester.edu

This PDF file includes:

Figs. S1 to S6
Table S1
Captions for Databases S1 to S4

Other supplementary materials for this manuscript include the following:

Databases S1 to S4

Table S1. Results of Linear Model

Antigen:	gp120					
Isotype:	IgG			IgG3		
Group	Mag	$t_{1/2}$	AUC	Mag	$t_{1/2}$	AUC
094-T2	2876.2	61.5	471.6	NA	NA	NA
094-T3	474.7	88.7	110.8	17.7	92.0	4.3
097-T1	12914.2	35.7	1231.0	167.3	37.3	16.7
097-T2	13425.0	31.8	1139.4	217.1	30.2	17.5
105-T2	7958.9	39.3	836.3	62.5	47.6	7.9
105-T3	7178.8	39.2	752.7	109.2	49.3	14.4
105-T4	5952.8	49.3	783.1	32.1	66.3	5.7
205-T1+T3	1564.9	51.5	215.2	54.5	44.2	6.4
205-T4	2014.9	69.6	372.9	61.7	66.0	10.8

Antigen:	gp140					
Isotype:	IgG			IgG3		
Group	Mag	$t_{1/2}$	AUC	Mag	$t_{1/2}$	AUC
094-T1	10548.5	106.9	2921.6	461.7	49.6	61.1
094-T2	15866.3	212.9	7468.1	246.0	99.3	63.8
094-T3	10386.2	139.3	3602.7	422.1	81.9	91.5
097-T1	15170.1	35.9	1456.5	228.9	37.9	23.2
097-T2	14207.3	34.0	1291.3	411.3	31.8	35.0
105-T2	14630.6	36.1	1412.1	190.0	37.5	19.1
105-T3	15967.5	40.5	1726.3	305.5	40.7	33.3
105-T4	18166.2	47.4	2299.1	53.4	59.8	8.5
205-T1+T3	3432.5	159.1	1320.2	589.9	41.1	64.7
205-T4	10217.2	147.7	3712.5	518.5	61.4	84.8

Antigen:	gp41					
Isotype:	IgG			IgG3		
Group	Mag	$t_{1/2}$	AUC	Mag	$t_{1/2}$	AUC
094-T1	10620.6	280.0	5858.0	3155.4	43.3	365.1
094-T2	12731.4	751.1	10026.4	1928.0	98.2	494.6
094-T3	14047.2	354.0	8670.2	3215.3	67.5	577.4
097-T1	7688.5	41.0	842.1	NA	NA	NA
097-T2	6619.9	36.5	646.0	NA	NA	NA
205-T1+T3	12401.7	100.1	3239.0	4003.5	39.3	420.1
205-T4	18975.3	134.2	6383.5	2115.9	59.5	335.6

Antigen:	V1V2					
Isotype:	IgG					
Group	Mag	$t_{1/2}$	AUC	Mag	$t_{1/2}$	AUC
094-T2	581.5220252	87.57874202	134.1696624			
097-T1	5482.253641	33.57044618	491.6893165			
097-T2	5402.746704	28.38733234	409.7501799			
105-T2	4440.343136	32.29525227	383.1174838			
105-T3	5384.144721	30.29375234	435.7613471			
105-T4	7641.737557	40.68918573	830.6306821			

Results of linear model: estimated mean peak magnitudes (MFI), estimated half lives (days), and time-averaged mean response (MFI) of IgG and IgG3 responses to gp120, gp140, gp41 and V1V2 region. Refer to table 1 for the specific strategies of each trial. See Fig 1 and Fig 2 for standard errors. NAs reflect trial arm and antigen combination for which modeling was not possible due to insufficient data or non-decreasing response. No models were run for V1V2 IgG3.

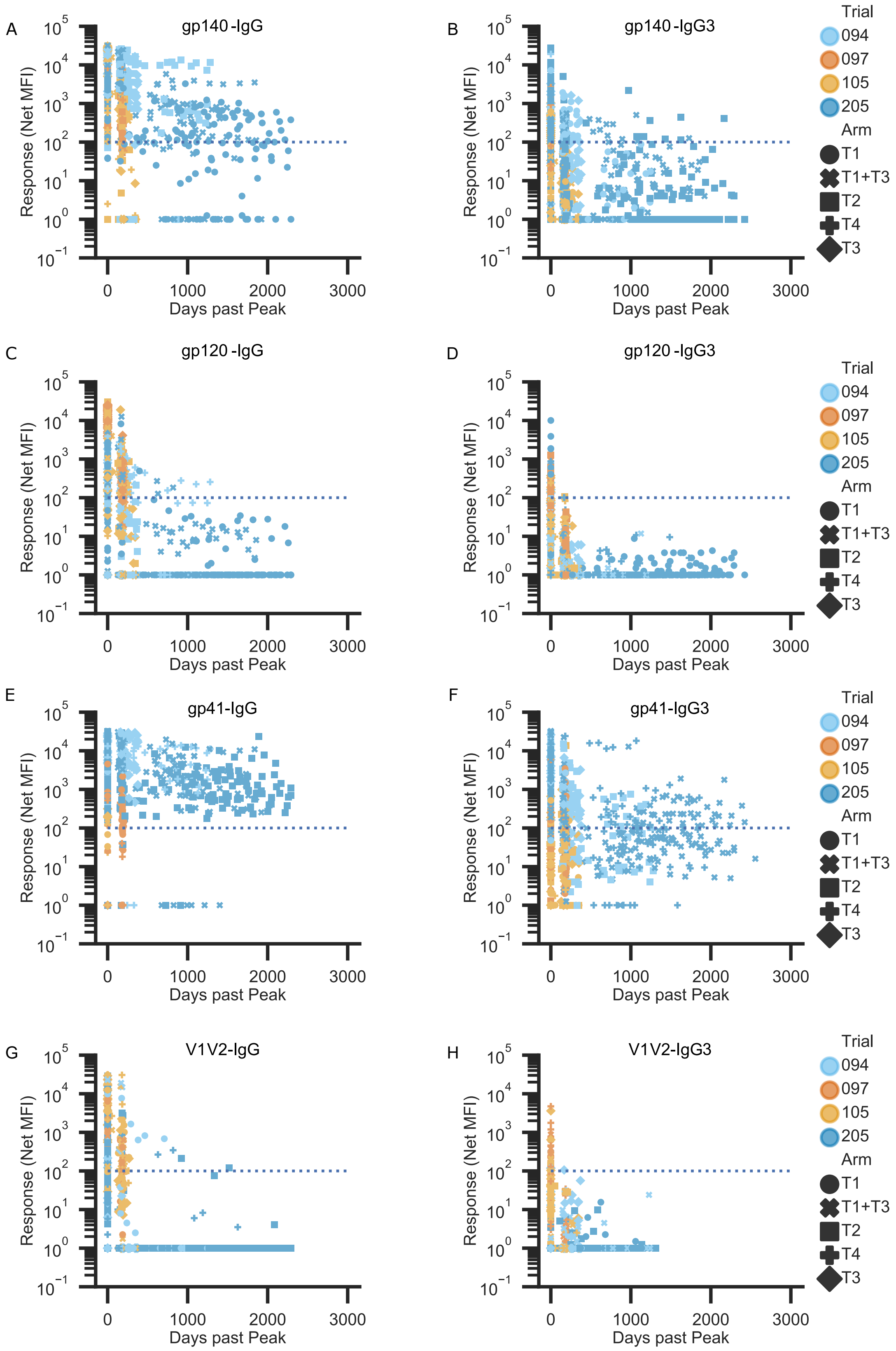

Fig. S1. Delta MFI from binding antibody multiplex assay to IgG against (A) gp140, (C) gp120, (E) gp41, (G) V1V2 region or IgG3 against (B) gp140, (D) gp120, (F) gp41, (H) V1V2 region. Data displayed starts at the peak response (2 weeks post vaccination) as day 0.

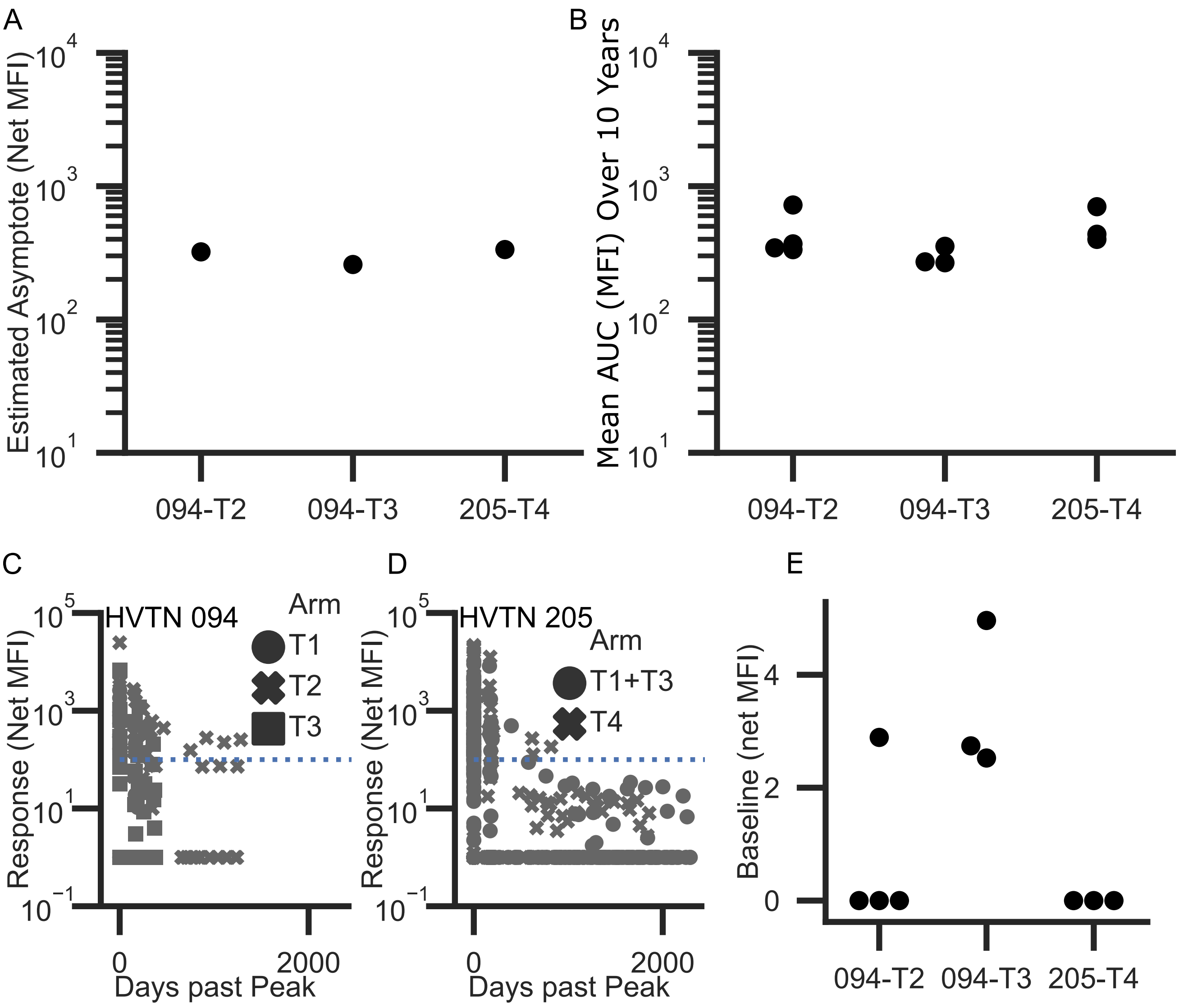

Fig. S2. gp120 antibody levels persists at high levels for up to a decade in MVA-boosted regimens. Estimated asymptote and time-average antibody response are shown for IgG responses to gp120. IgG response levels for all individuals in (C) HVTN 094 and (D) HVTN 205 trials against gp120 are shown. (E) (Pre-vaccination) Baseline response against gp140 for individuals included in model are shown. Participants who have gp120 specific antibody levels >100 Net MFI after 180 days are included. Due to the limited number of participants that satisfied above criteria only point estimates could be calculated.

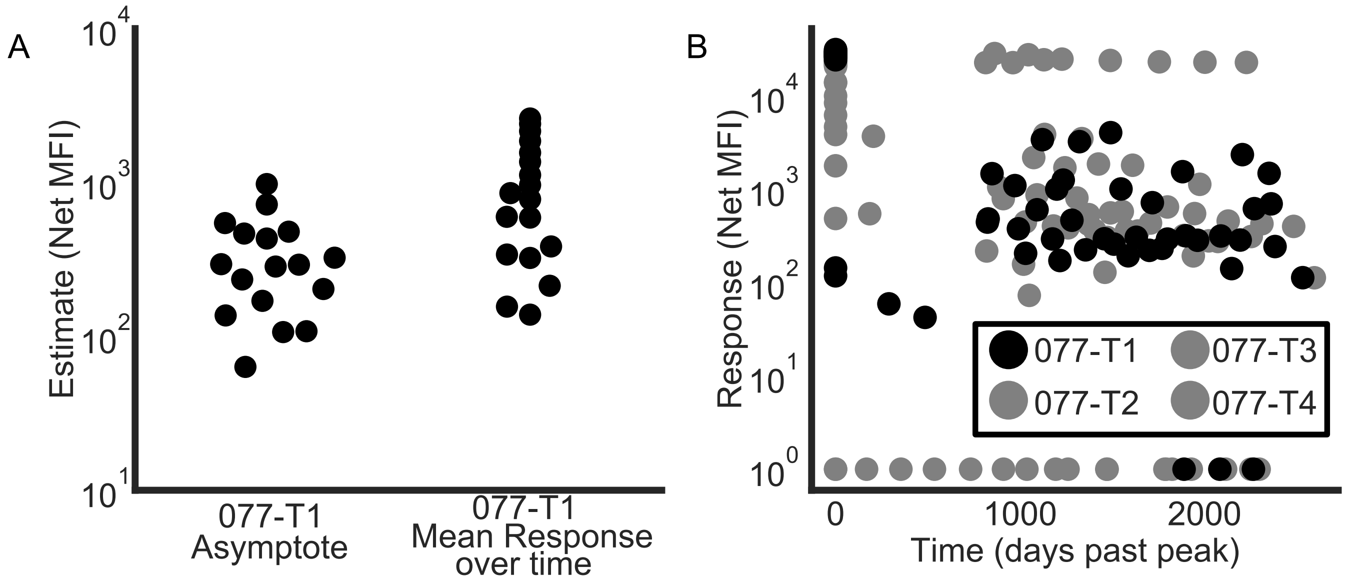

Fig. S3. Antibody responses can persist at high levels for up to a decade after Adenovirus-vectored vaccination. (A) Estimated asymptote and time-average mean antibody response are shown for HVTN 077-T1 IgG responses to gp140. Only regimens with participants who still had levels of IgG to gp140 >100 Net MFI after 180 days are included. (B) IgG response levels for all individuals in HVTN 077 against gp140 are shown. 077-T1 consists of an Ad35 prime with an Ad5 boost while 077-T2,3 and 4 consist of a DNA prime with either an Ad5 (T2) or Ad35 (T3, T4) boost. HVTN 077 utilizes a gp140 envelope sequence as the immunogen.

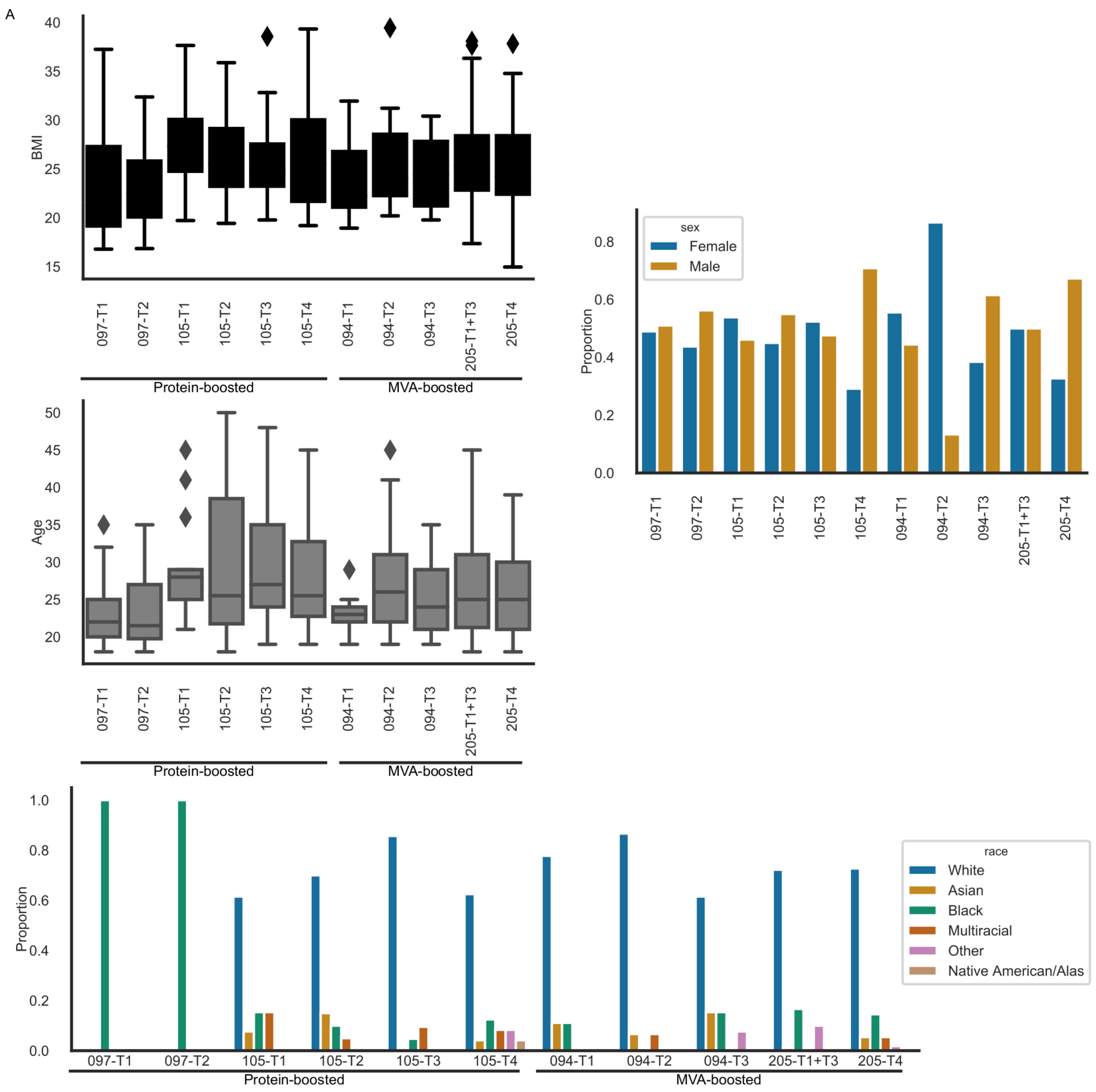

Fig. S4. Distribution of demographic variables. (A) BMI, (B) age, (C) race, and (D) sex shown for each group assessed in this study. Demographics are shown for participants included in linear model for any antigen/isotype in our study.

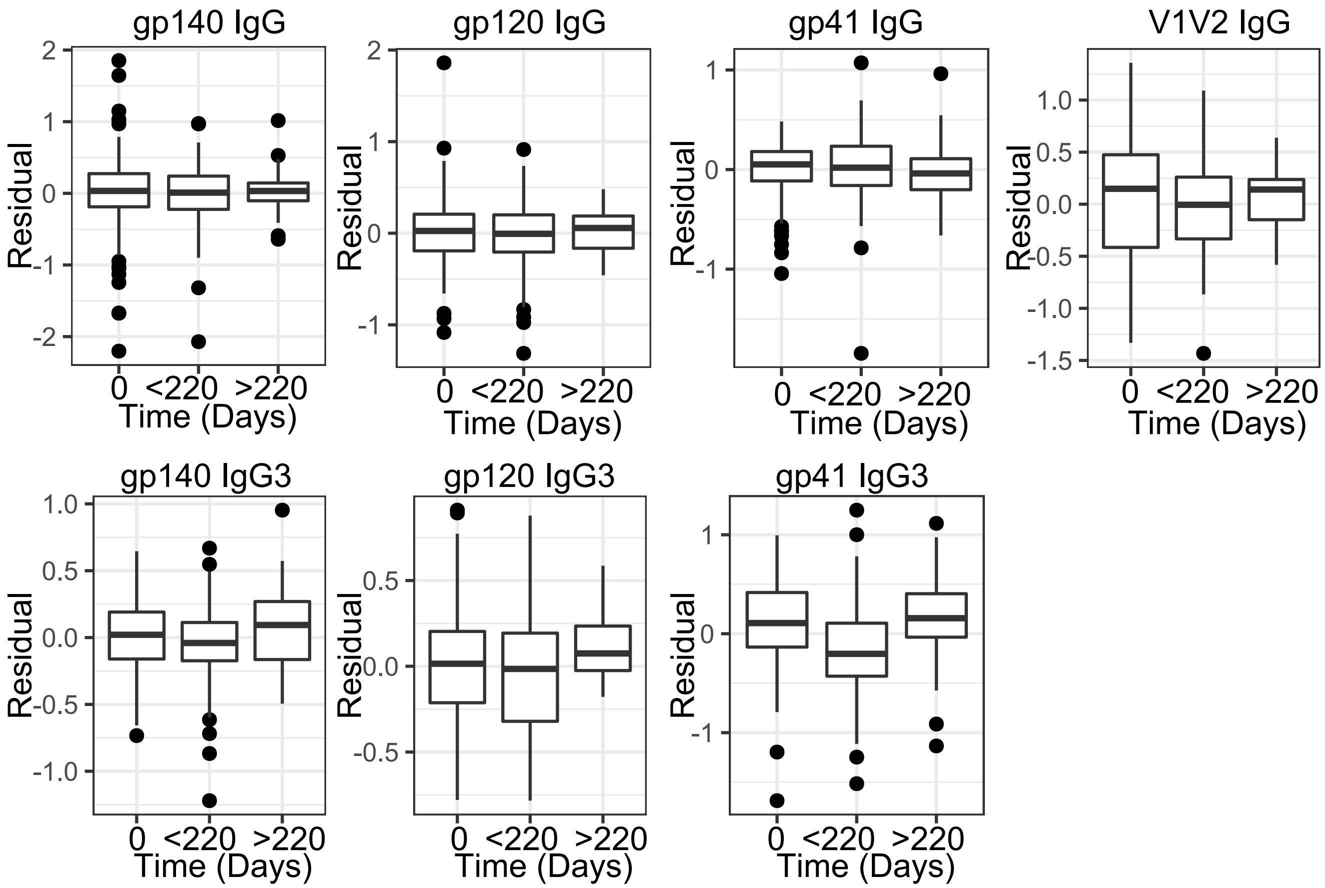

Fig. S5. Residuals are well distributed across time. Residuals (Pearson) for all linear models across time. Each boxplot is labeled with the antigen-isotype combination and divided into three boxes: peak, six months (<220), and after six months (>220).

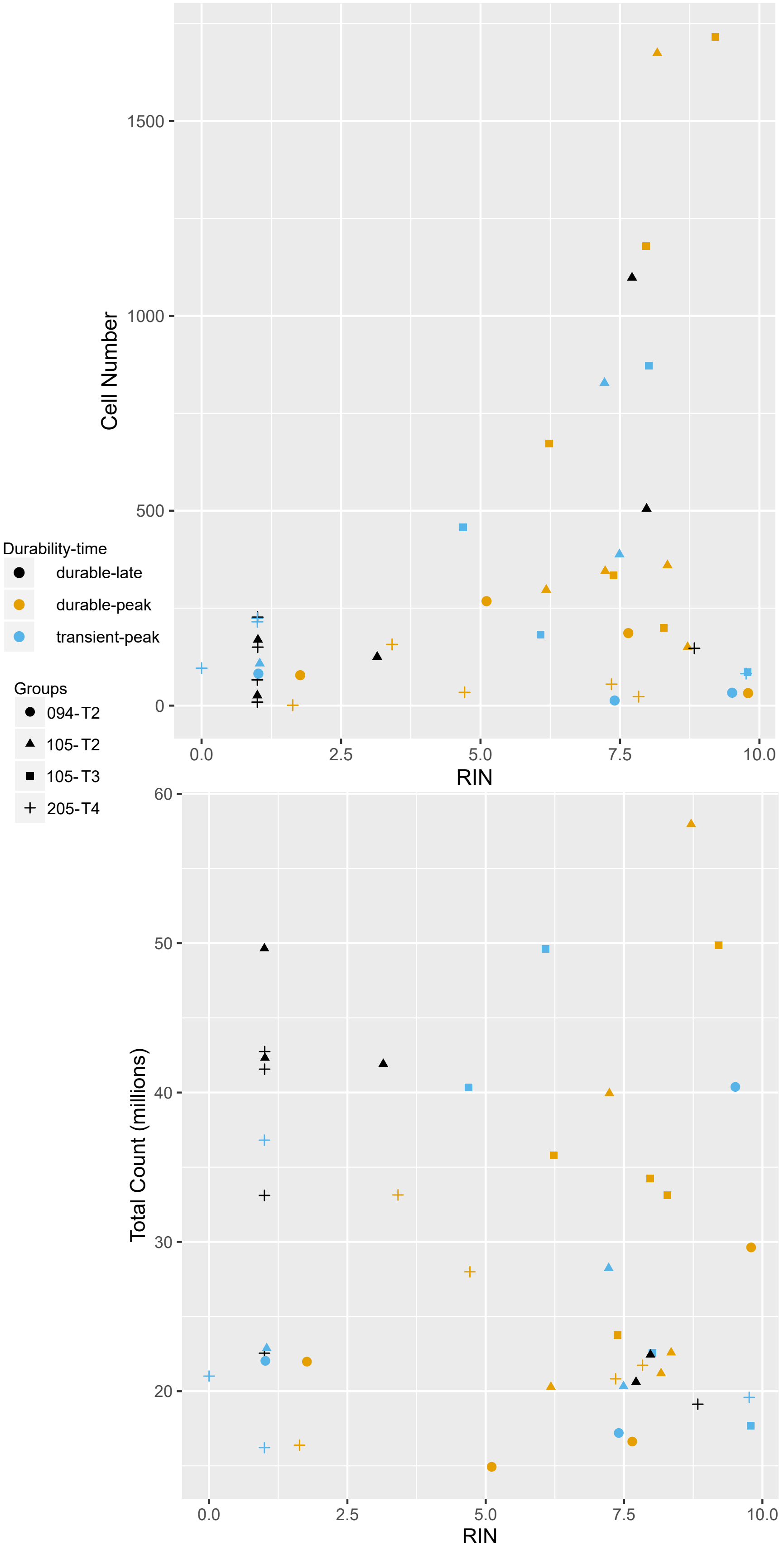

Fig. S6. Transcriptomics quality control. (top) Cell number vs RNA Integrity Number (RIN) and (bottom) Total RNA count vs RIN with trials shown by shape and durability as well as time shown with color. Late indicates samples collected six months post-peak.

Additional data table S1 (DE_genes.csv)
Differentially expressed genes identified between protein and MVA.

Additional data table S2 (GSEA.csv)
Results of gene set enrichment analysis to show pathway-level differences between protein and MVA.

Additional data table S3 (BONITA_results.csv)
Results of BONITA pathway analysis. Log10 p-values provided for several contrasts.

Additional data table S4 (correlates.csv)
Genes correlated with half life.
